# Single-cell transcriptomic landscape of human blood cells

**DOI:** 10.1093/nsr/nwaa180

**Published:** 2020-08-24

**Authors:** Xiaowei Xie, Mengyao Liu, Yawen Zhang, Bingrui Wang, Caiying Zhu, Chenchen Wang, Qing Li, Yingying Huo, Jiaojiao Guo, Changlu Xu, Linping Hu, Aiming Pang, Shihui Ma, Lina Wang, Wenbin Cao, Shulian Chen, Qiuling Li, Sudong Zhang, Xueying Zhao, Wen Zhou, Hongbo Luo, Guoguang Zheng, Erlie Jiang, Sizhou Feng, Lixiang Chen, Lihong Shi, Hui Cheng, Sha Hao, Ping Zhu, Tao Cheng

**Affiliations:** State Key Laboratory of Experimental Hematology and National Clinical Research Center for Blood Diseases, Institute of Hematology and Blood Diseases Hospital, Chinese Academy of Medical Sciences & Peking Union Medical College, Tianjin 300020, China; Center for Stem Cell Medicine and Department of Stem Cell & Regenerative Medicine, Chinese Academy of Medical Sciences and Peking Union Medical College, Tianjin 300020, China; State Key Laboratory of Experimental Hematology and National Clinical Research Center for Blood Diseases, Institute of Hematology and Blood Diseases Hospital, Chinese Academy of Medical Sciences & Peking Union Medical College, Tianjin 300020, China; Center for Stem Cell Medicine and Department of Stem Cell & Regenerative Medicine, Chinese Academy of Medical Sciences and Peking Union Medical College, Tianjin 300020, China; State Key Laboratory of Experimental Hematology and National Clinical Research Center for Blood Diseases, Institute of Hematology and Blood Diseases Hospital, Chinese Academy of Medical Sciences & Peking Union Medical College, Tianjin 300020, China; Center for Stem Cell Medicine and Department of Stem Cell & Regenerative Medicine, Chinese Academy of Medical Sciences and Peking Union Medical College, Tianjin 300020, China; State Key Laboratory of Experimental Hematology and National Clinical Research Center for Blood Diseases, Institute of Hematology and Blood Diseases Hospital, Chinese Academy of Medical Sciences & Peking Union Medical College, Tianjin 300020, China; Center for Stem Cell Medicine and Department of Stem Cell & Regenerative Medicine, Chinese Academy of Medical Sciences and Peking Union Medical College, Tianjin 300020, China; State Key Laboratory of Experimental Hematology and National Clinical Research Center for Blood Diseases, Institute of Hematology and Blood Diseases Hospital, Chinese Academy of Medical Sciences & Peking Union Medical College, Tianjin 300020, China; Center for Stem Cell Medicine and Department of Stem Cell & Regenerative Medicine, Chinese Academy of Medical Sciences and Peking Union Medical College, Tianjin 300020, China; State Key Laboratory of Experimental Hematology and National Clinical Research Center for Blood Diseases, Institute of Hematology and Blood Diseases Hospital, Chinese Academy of Medical Sciences & Peking Union Medical College, Tianjin 300020, China; Center for Stem Cell Medicine and Department of Stem Cell & Regenerative Medicine, Chinese Academy of Medical Sciences and Peking Union Medical College, Tianjin 300020, China; State Key Laboratory of Experimental Hematology and National Clinical Research Center for Blood Diseases, Institute of Hematology and Blood Diseases Hospital, Chinese Academy of Medical Sciences & Peking Union Medical College, Tianjin 300020, China; Center for Stem Cell Medicine and Department of Stem Cell & Regenerative Medicine, Chinese Academy of Medical Sciences and Peking Union Medical College, Tianjin 300020, China; State Key Laboratory of Experimental Hematology and National Clinical Research Center for Blood Diseases, Institute of Hematology and Blood Diseases Hospital, Chinese Academy of Medical Sciences & Peking Union Medical College, Tianjin 300020, China; Center for Stem Cell Medicine and Department of Stem Cell & Regenerative Medicine, Chinese Academy of Medical Sciences and Peking Union Medical College, Tianjin 300020, China; Cancer Research Institute, School of Basic Medical Science, Central South University, Changsha 410078, China; State Key Laboratory of Experimental Hematology and National Clinical Research Center for Blood Diseases, Institute of Hematology and Blood Diseases Hospital, Chinese Academy of Medical Sciences & Peking Union Medical College, Tianjin 300020, China; Center for Stem Cell Medicine and Department of Stem Cell & Regenerative Medicine, Chinese Academy of Medical Sciences and Peking Union Medical College, Tianjin 300020, China; State Key Laboratory of Experimental Hematology and National Clinical Research Center for Blood Diseases, Institute of Hematology and Blood Diseases Hospital, Chinese Academy of Medical Sciences & Peking Union Medical College, Tianjin 300020, China; Center for Stem Cell Medicine and Department of Stem Cell & Regenerative Medicine, Chinese Academy of Medical Sciences and Peking Union Medical College, Tianjin 300020, China; State Key Laboratory of Experimental Hematology and National Clinical Research Center for Blood Diseases, Institute of Hematology and Blood Diseases Hospital, Chinese Academy of Medical Sciences & Peking Union Medical College, Tianjin 300020, China; Center for Stem Cell Medicine and Department of Stem Cell & Regenerative Medicine, Chinese Academy of Medical Sciences and Peking Union Medical College, Tianjin 300020, China; State Key Laboratory of Experimental Hematology and National Clinical Research Center for Blood Diseases, Institute of Hematology and Blood Diseases Hospital, Chinese Academy of Medical Sciences & Peking Union Medical College, Tianjin 300020, China; Center for Stem Cell Medicine and Department of Stem Cell & Regenerative Medicine, Chinese Academy of Medical Sciences and Peking Union Medical College, Tianjin 300020, China; State Key Laboratory of Experimental Hematology and National Clinical Research Center for Blood Diseases, Institute of Hematology and Blood Diseases Hospital, Chinese Academy of Medical Sciences & Peking Union Medical College, Tianjin 300020, China; Center for Stem Cell Medicine and Department of Stem Cell & Regenerative Medicine, Chinese Academy of Medical Sciences and Peking Union Medical College, Tianjin 300020, China; State Key Laboratory of Experimental Hematology and National Clinical Research Center for Blood Diseases, Institute of Hematology and Blood Diseases Hospital, Chinese Academy of Medical Sciences & Peking Union Medical College, Tianjin 300020, China; Center for Stem Cell Medicine and Department of Stem Cell & Regenerative Medicine, Chinese Academy of Medical Sciences and Peking Union Medical College, Tianjin 300020, China; State Key Laboratory of Experimental Hematology and National Clinical Research Center for Blood Diseases, Institute of Hematology and Blood Diseases Hospital, Chinese Academy of Medical Sciences & Peking Union Medical College, Tianjin 300020, China; Center for Stem Cell Medicine and Department of Stem Cell & Regenerative Medicine, Chinese Academy of Medical Sciences and Peking Union Medical College, Tianjin 300020, China; State Key Laboratory of Experimental Hematology and National Clinical Research Center for Blood Diseases, Institute of Hematology and Blood Diseases Hospital, Chinese Academy of Medical Sciences & Peking Union Medical College, Tianjin 300020, China; Center for Stem Cell Medicine and Department of Stem Cell & Regenerative Medicine, Chinese Academy of Medical Sciences and Peking Union Medical College, Tianjin 300020, China; State Key Laboratory of Experimental Hematology and National Clinical Research Center for Blood Diseases, Institute of Hematology and Blood Diseases Hospital, Chinese Academy of Medical Sciences & Peking Union Medical College, Tianjin 300020, China; Center for Stem Cell Medicine and Department of Stem Cell & Regenerative Medicine, Chinese Academy of Medical Sciences and Peking Union Medical College, Tianjin 300020, China; State Key Laboratory of Experimental Hematology and National Clinical Research Center for Blood Diseases, Institute of Hematology and Blood Diseases Hospital, Chinese Academy of Medical Sciences & Peking Union Medical College, Tianjin 300020, China; Center for Stem Cell Medicine and Department of Stem Cell & Regenerative Medicine, Chinese Academy of Medical Sciences and Peking Union Medical College, Tianjin 300020, China; Cancer Research Institute, School of Basic Medical Science, Central South University, Changsha 410078, China; State Key Laboratory of Experimental Hematology and National Clinical Research Center for Blood Diseases, Institute of Hematology and Blood Diseases Hospital, Chinese Academy of Medical Sciences & Peking Union Medical College, Tianjin 300020, China; Center for Stem Cell Medicine and Department of Stem Cell & Regenerative Medicine, Chinese Academy of Medical Sciences and Peking Union Medical College, Tianjin 300020, China; State Key Laboratory of Experimental Hematology and National Clinical Research Center for Blood Diseases, Institute of Hematology and Blood Diseases Hospital, Chinese Academy of Medical Sciences & Peking Union Medical College, Tianjin 300020, China; Center for Stem Cell Medicine and Department of Stem Cell & Regenerative Medicine, Chinese Academy of Medical Sciences and Peking Union Medical College, Tianjin 300020, China; State Key Laboratory of Experimental Hematology and National Clinical Research Center for Blood Diseases, Institute of Hematology and Blood Diseases Hospital, Chinese Academy of Medical Sciences & Peking Union Medical College, Tianjin 300020, China; Center for Stem Cell Medicine and Department of Stem Cell & Regenerative Medicine, Chinese Academy of Medical Sciences and Peking Union Medical College, Tianjin 300020, China; State Key Laboratory of Experimental Hematology and National Clinical Research Center for Blood Diseases, Institute of Hematology and Blood Diseases Hospital, Chinese Academy of Medical Sciences & Peking Union Medical College, Tianjin 300020, China; Center for Stem Cell Medicine and Department of Stem Cell & Regenerative Medicine, Chinese Academy of Medical Sciences and Peking Union Medical College, Tianjin 300020, China; School of Life Sciences, Zhengzhou University, Zhengzhou 450001, China; State Key Laboratory of Experimental Hematology and National Clinical Research Center for Blood Diseases, Institute of Hematology and Blood Diseases Hospital, Chinese Academy of Medical Sciences & Peking Union Medical College, Tianjin 300020, China; Center for Stem Cell Medicine and Department of Stem Cell & Regenerative Medicine, Chinese Academy of Medical Sciences and Peking Union Medical College, Tianjin 300020, China; State Key Laboratory of Experimental Hematology and National Clinical Research Center for Blood Diseases, Institute of Hematology and Blood Diseases Hospital, Chinese Academy of Medical Sciences & Peking Union Medical College, Tianjin 300020, China; Center for Stem Cell Medicine and Department of Stem Cell & Regenerative Medicine, Chinese Academy of Medical Sciences and Peking Union Medical College, Tianjin 300020, China; State Key Laboratory of Experimental Hematology and National Clinical Research Center for Blood Diseases, Institute of Hematology and Blood Diseases Hospital, Chinese Academy of Medical Sciences & Peking Union Medical College, Tianjin 300020, China; Center for Stem Cell Medicine and Department of Stem Cell & Regenerative Medicine, Chinese Academy of Medical Sciences and Peking Union Medical College, Tianjin 300020, China; State Key Laboratory of Experimental Hematology and National Clinical Research Center for Blood Diseases, Institute of Hematology and Blood Diseases Hospital, Chinese Academy of Medical Sciences & Peking Union Medical College, Tianjin 300020, China; Center for Stem Cell Medicine and Department of Stem Cell & Regenerative Medicine, Chinese Academy of Medical Sciences and Peking Union Medical College, Tianjin 300020, China; State Key Laboratory of Experimental Hematology and National Clinical Research Center for Blood Diseases, Institute of Hematology and Blood Diseases Hospital, Chinese Academy of Medical Sciences & Peking Union Medical College, Tianjin 300020, China; Center for Stem Cell Medicine and Department of Stem Cell & Regenerative Medicine, Chinese Academy of Medical Sciences and Peking Union Medical College, Tianjin 300020, China

**Keywords:** human, hematopoietic cells, lncRNAs, transcription factor, single-cell RNA-seq

## Abstract

High throughput single-cell RNA-seq has been successfully implemented to dissect the cellular and molecular features underlying hematopoiesis. However, an elaborate and comprehensive transcriptome reference of the whole blood system is lacking. Here, we profiled the transcriptomes of 7551 human blood cells representing 32 immunophenotypic cell types, including hematopoietic stem cells, progenitors and mature blood cells derived from 21 healthy donors. With high sequencing depth and coverage, we constructed a single-cell transcriptional atlas of blood cells (ABC) on the basis of both protein-coding genes and long noncoding RNAs (lncRNAs), and showed a high consistence between them. Notably, putative lncRNAs and transcription factors regulating hematopoietic cell differentiation were identified. While common transcription factor regulatory networks were activated in neutrophils and monocytes, lymphoid cells dramatically changed their regulatory networks during differentiation. Furthermore, we showed a subset of nucleated erythrocytes actively expressing immune signals, suggesting the existence of erythroid precursors with immune functions. Finally, a web portal offering transcriptome browsing and blood cell type prediction has been established. Thus, our work provides a transcriptional map of human blood cells at single-cell resolution, thereby offering a comprehensive reference for the exploration of physiological and pathological hematopoiesis.

## INTRODUCTION

The hematopoietic system, mainly derived from a pool of hematopoietic stem/progenitor cells (HSPCs), continuously generates erythrocytes/megakaryocytes, myeloid cells and lymphocytes and plays vital roles throughout the whole human lifespan. Dysregulation of hematopoiesis may give rise to various diseases such as immunodeficiency and blood cancers [1–3]. In recent years, single-cell sequencing techniques have facilitated the exploration of cellular and molecular heterogeneity during hematopoietic cell differentiation [4]. Research investigating hematopoietic progenitor cells has highlighted the differentiation hierarchy of early hematopoiesis [5–7]. In contrast, lineage^−^CD34^+^CD38^−^ cells have been shown to be a transcriptional continuum of low-primed undifferentiated hematopoietic stem and progenitor cells [8]. Although these studies have considerably advanced our perception of hematopoiesis, a systematic view of hematopoietic cell differentiation based on multiple individuals and blood cell types by deep single-cell RNA sequencing has been lacking.

LncRNAs function as crucial regulators during hematopoietic cell differentiation and development [9], including hematopoietic stem cell (HSC) differentiation, erythropoiesis and the development of B and T lymphocytes [10–13]. Recently, lncRNAs were comprehensively defined at the single-cell level and displayed highly lineage-specific and dynamic expression during human HSPC differentiation [14]. The single-cell lncRNA landscape of embryonic hematopoietic stem cells was also profiled and lncRNA (H19) was recognized to be pivotal for HSC emergence [15]. Nevertheless, the full repertoire of lncRNAs in human blood cells has not been elucidated.

In this study, we constructed a comprehensive transcriptome reference of human blood cells (that is atlas of blood cells, ABC), by performing deep sequencing of over 7000 single cells, representing 32 immunophenotypic cell types from 21 healthy donors. The hematopoietic hierarchy was dissected based on both protein-coding genes and lncRNAs. Of note, we elaborated the dynamic regulatory networks and differentiation trajectories for HSPCs and each lineage. This work contributes to a comprehensive understanding of the molecular dynamics during lineage differentiation, and provides valuable transcriptome references for hematopoiesis under both homeostasis and disease.

## RESULTS

### Human hematopoietic transcriptome reference

**Figure 1.**
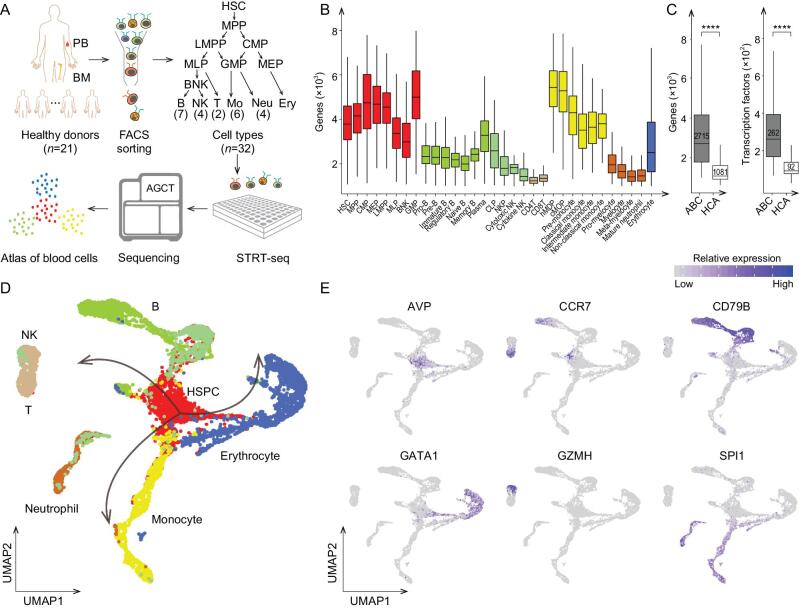
Transcriptome reference of human blood cells. (A) Scheme of the experimental design. (B) Box plot shows the detected gene number for 32 immunophenotypic cell types. Colors indicate cell types. (C) Atlas of Blood Cells (ABC) detects more genes and transcription factors than the bone marrow samples from Human Cell Atlas (HCA) (*P* values < 2.2e^−16^, Wilcoxon test). (D) Transcriptional atlas of 7551 human hematopoietic cells by UMAP. Colors indicate cell populations and arrows show the differentiation trajectories of lymphoid, neutrophil/monocyte and erythroid lineages. (E) UMAP displays of transcription activities for hematopoiesis-related genes (*AVP*, *CD79B*, *GZMH*, *CCR7*, *SPI1* and *GATA1*, respectively, for HSPCs, B cells, NK cells, T cells, neutrophil/monocytes and erythrocytes).

To establish a comprehensive transcriptome reference of the human blood system, we profiled the transcriptomes of bone marrow and peripheral blood derived hematopoietic cells from 21 healthy adult donors, by combining fluorescence activated cell sorting (FACS) of single cells for 32 well-defined cell types and a Single-Cell Tagged Reverse Transcription RNA Sequencing (STRT-seq) strategy (Methods). Specifically, we harvested bone marrow derived progenitors and differentiated cells (including CD34^+^ HSPCs, B cells, NK cells, T cells, monocytes, neutrophils and erythrocytes), together with peripheral blood derived differentiated cells including regulatory B, naive B, memory B, cytotoxic NK, cytokine NK and T cells (Supplementary Fig. 1). In total, 7551 single cells were profiled to construct the transcriptional atlas of human hematopoietic cells (Fig. 1A and Supplementary Fig. 2A). By limiting the number of single cells mixed in each library and increasing the sequencing depth, we were able to detect on average ∼3000 protein-coding genes per single cell. This high quality of transcriptome data ensured an accurate construction of the hematopoietic hierarchy with more gene expression details. HSPCs and monocytes expressed the highest number of genes, whereas NK cells, T cells and neutrophils were relatively transcriptionally quiescent (Fig. 1B). Notably, compared with the data generated using 10X Genomics sequencing of bone marrow derived nucleated cells [7], our data enabled detection of more genes and transcription factors for in-depth analysis (*P* values < 2.2e^−16^, Wilcoxon test, Fig. 1C), and were particularly superior in detecting low-abundance genes (Supplementary Fig. 2B).

We first integrated the single-cell transcriptome profiles of all hematopoietic cells, followed by dimension reduction and visualization by uniform manifold approximation and projection (UMAP). The overall differentiation trajectory of hematopoiesis was revealed by starting from HSPCs and branching toward lymphocytes (B cells, NK cells and T cells), myeloid cells (monocytes and neutrophils) and erythrocytes. In contrast to B cells, monocytes and erythrocytes, we found that NK cells, T cells and neutrophils lacked a continuous transcriptional transition from progenitors to differentiated cells, suggesting that these cells might acquire dramatic shifts in gene expression during maturation, or that there existed transition populations not captured by the known surface markers (Fig. 1D and Supplementary Fig. 2C). We observed that *AVP* was specifically expressed in HSCs and MPPs. *CD79B*, *GZMH* and *CCR7*, respectively, exhibited high specificity and expression in B cells, NK cells and T cells, while *SPI1* and *GATA1* were highly expressed in neutrophil/monocytes and erythrocytes, respectively (Fig. 1E), verifying well-known marker genes related to hematopoiesis in each cell type. By deep sequencing of multiple classical hematopoietic populations from more than 20 healthy donors, our single-cell transcriptional atlas of human blood cells provides valuable transcriptome references for studies of human physiological and pathological hematopoiesis.

### Regulatory networks underlie hematopoietic differentiation

**Figure 2.**
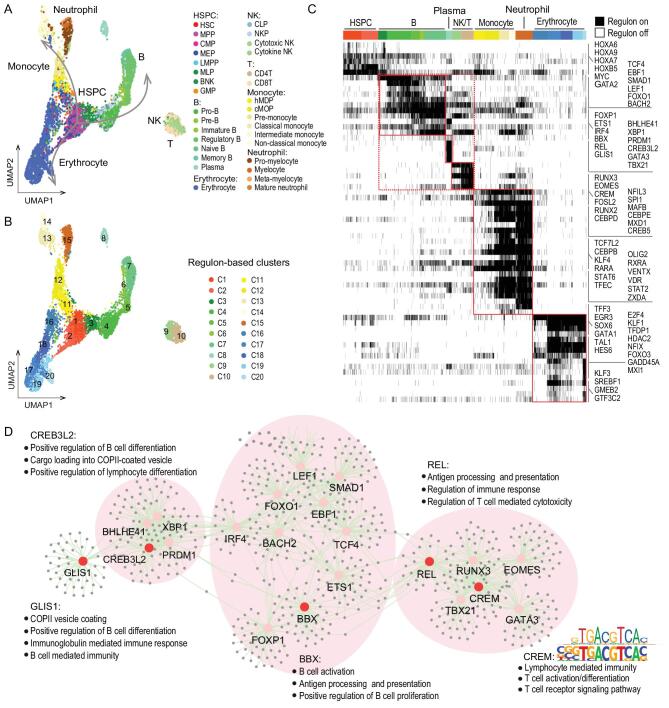
Transcription factor regulatory networks underlie hematopoiesis. (A) UMAP displays the distributions of 32 immunophenotypic cell types based on regulons. Colors indicate cell types and arrows reflect the differentiation trajectories of lymphoid, neutrophil/monocyte and erythroid lineages. (B) 20 regulatory clusters determined by Seurat package. C1 to C20 indicates the regulatory clusters. (C) Heat map displays the on/off states of cluster-specific regulons. Black represents when the network is activated, and white represents when the network is inactivated. Red boxes emphasize the regulons for identical cell types or lineages. (D) Cytoscape shows the regulatory networks comprising transcription factors and their target genes underlying the lymphoid lineage. Edges connect transcription factor-target gene pairs while nodes represent genes. Transcription factors are displayed in larger font size and red color highlights the novel ones. Enriched biological processes and motif sequences (above: annotated motif from JASPAR; below: enriched motif) of target genes for novel transcription factors are shown. Shadows highlight the cell-type specific sub-networks.

To resolve the transcription factor regulatory networks (regulons) that underlie hematopoietic differentiation, regulon activity scores (RASs) were calculated for transcription factors in all single cells using SCENIC [16], which were then submitted to construct the regulatory atlas of human blood cells. We observed that the overall differentiation trajectory of hematopoiesis revealed by regulons coincided with that revealed by single-cell transcriptomes (Fig. 2A). In addition, SPRING [17] was used to visualize the hematopoietic hierarchy deduced from regulons and recapitulate the trajectory of lineage branches (Supplementary Fig. 3A). Single cells were distributed according to their cell types instead of according to the donors, suggesting no batch effects or individual diversities in terms of regulons related to hematopoiesis. Hematopoietic cells were grouped into 20 regulatory clusters by unsupervised clustering, termed as C1 to C20 hereafter, and each showed activation of highly specific sets of regulons (Fig. 2B). In particular, HOX genes were activated and co-regulated in C1/C2, which were mainly composed of HSPCs. *TCF4*, *EBF1* and *LEF1* showed high activities in C3 to C7 representing B cells, *PRDM1* and *XBP1* were activated in plasma cells, while *GATA3* and *TBX21* were activated in NK/T cells. *CEBP* and *SPI1* exhibited high activities in the neutrophil/monocyte

lineage, while *GATA1* and *KLF1* were activated in the erythroid lineage (Fig. 2C and Supplementary Fig. 3B). Remarkably, monocytes and neutrophils shared the majority of regulons unique to myeloid lineage, while lymphocytes were preferentially regulated by cell type specific sub-networks highlighted by the regulon shifts during B cell maturation (Fig. 2C).

We next identified 23 novel regulons activated in each lineage by comparing with previously reported canonical transcription factors. To further confirm the regulatory functions of novel transcription factors, gene ontology (GO) enrichment and motif analysis of their target genes were performed accordingly. We found that enriched GO terms for *BBX*, *REL*, *CREM*, *CREB3L2* and *GLIS1* were associated with the activation/differentiation of

 

lymphocytes (Fig. 2D). *RXRA*, *STAT2*, *STAT6*, *TFF3*, *TFEC*, *EGR3*, *OLIG2*, *VDR*, *VENTX* and *ZXDA* were targeting neutrophil/granulocyte related genes, while *FOXO3*, *NFIX*, *KLF3*, *MXI1*, *GMEB2*, *GTF3C2*, *SREBF1* and *GADD45A* targeted genes were highly related to biological processes such as erythrocyte/oxygen/iron ion (Supplementary Fig. 3C and D). Additionally, the enriched sequences on the promoter region of target genes for transcription factors like *CREM*, *TFF3* and *NFIX* were consistent with their annotated motifs, suggesting that these newly identified transcription factors might regulate hematopoiesis in cooperating with canonical networks (Fig. 2D and Supplementary Fig. 3C and D).

### Landscapes of long non-coding RNAs

**Figure 3.**
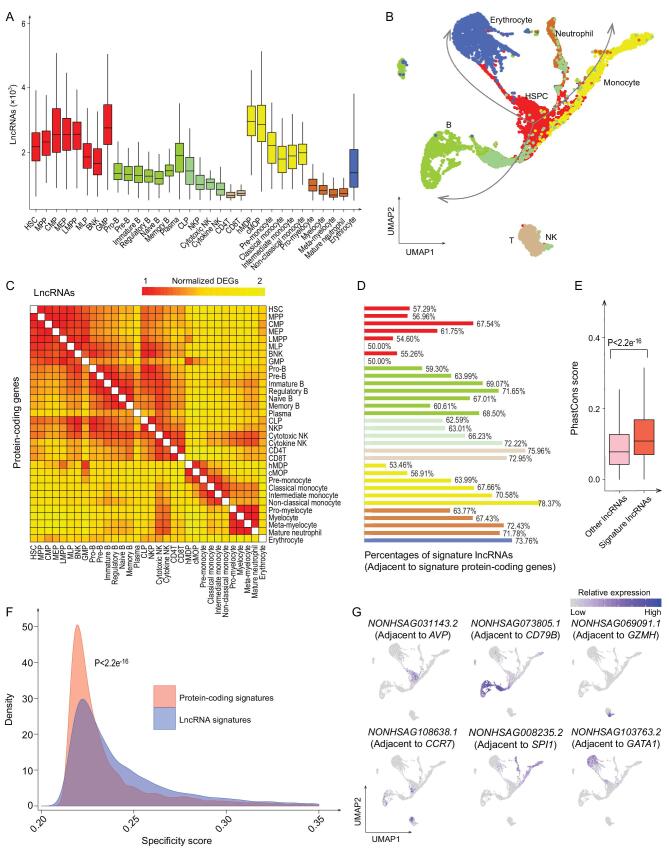
Reconstruction of hematopoietic hierarchy by using lncRNAs. (A) Box plot shows the detected lncRNA number for 32 immunophenotypic cell types. Colors indicate cell types. (B) Transcriptional atlas of 7192 human hematopoietic cells based on lncRNAs by UMAP. (C) Heat map shows the scaled DEG numbers between 32 immunophenotypic cell types based on protein-coding genes (the lower triangle) and lncRNAs (the upper triangle). (D) Bar plot shows the proportion of signature lncRNAs adjacent to signature protein-coding genes for each immunophenotypic cell type. (E and F) Signature lncRNAs show higher PhastCons conservation scores (E) and higher cell specificity (F) (*P* values < 2.2e^−16^, Wilcoxon test). (G) UMAP displays of transcription activities for hematopoiesis-related lncRNAs such as *NONHSAG031143.2*, *NONHSAG073805.1*, *NONHSAG069091.1*, *NONHSAG108638.1*, *NONHSAG008235.2* and *NONHSAG103763.2* (respectively adjacent to *AVP*, *CD79B*, *GZMH*, *CCR7*, *SPI1* and *GATA1*).

lncRNAs play crucial roles in hematopoietic differentiation and development. However, the expression spectrum of lncRNAs in hematopoietic cells at single-cell resolution has not been reported. Therefore, we sought to construct a transcriptional atlas of 7192 human blood cells based on lncRNAs by taking advantage of our deep sequencing datasets. On average, over 1700 lncRNAs were detected for 32 immunophenotypic cell types referred to the genome annotation from the NONCODE database [18]. Coincident with protein-coding genes, HSPCs and monocytes expressed the highest number of lncRNAs, whereas NK cells, T cells and neutrophils expressed the lowest number of lncRNAs (Fig. 3A). We showed that the hematopoietic differentiation trajectory reconstructed by only using lncRNAs was highly consistent with that revealed by protein-coding genes (Fig. 3B and Supplementary Fig. 4A). To further dissect the transcriptional heterogeneity, differentially expressed genes (DEGs) were calculated between any two immunophenotypic cell types for both protein-coding genes and lncRNAs. The normalized number of DEGs showed high agreement between lncRNAs and protein-coding genes (Fig. 3C). These results implicated that the dynamics of lncRNAs were capable of depicting the global hematopoietic hierarchy.

Next, we identified signature protein-coding genes and lncRNAs for each immunophenotypic cell type. We found that signature lncRNAs tended to associate with their adjacent signature protein-coding genes for more differentiated cell types, in contrast to progenitors (Fig. 3D). However, distal signature lncRNAs did not show significant change tendency during differentiation relative to adjacent signature lncRNAs (Supplementary Fig. 4B). Moreover, signature lncRNAs showed higher PhastCons conservation scores (from UCSC 100-way) and higher cell specificity (based on Jensen-Shannon Divergence, JSD) compared to background estimations (*P* values <2.2e^−16^, Wilcoxon test) (Fig. 3E and F), suggesting their potential functional roles. To further exploit the function of signature lncRNAs, their neighboring protein-coding genes with distances less than 5kb were scanned along the genome. Protein-coding genes near signature lncRNAs significantly overlapped with hematopoietic signatures [7,8] (Supplementary Fig. 4C). In particular, lncRNAs (*NONHSAG031143.2*, *NONHSAG073805.1*, *NONHSAG069091.1*, *NONHSAG108638.1*, *NONHSAG008235.2* and *NONHSAG103763.2*) adjacent to hematopoietic signatures (*AVP*, *CD79B*, *GZMH*, *CCR7*, *SPI1* and *GATA1*) were specifically highly expressed in HSPCs, B cells, NK cells, T cells, neutrophil/monocytes and erythrocytes, respectively (Fig. 3G). Altogether, lncRNAs specifically expressed in hematopoietic cells were identified at the single-cell level, and they tended to co-express with their adjacent signature protein-coding genes for more differentiated cells.

### Elaborate atlas for each hematopoietic cell population

**Figure 4.**
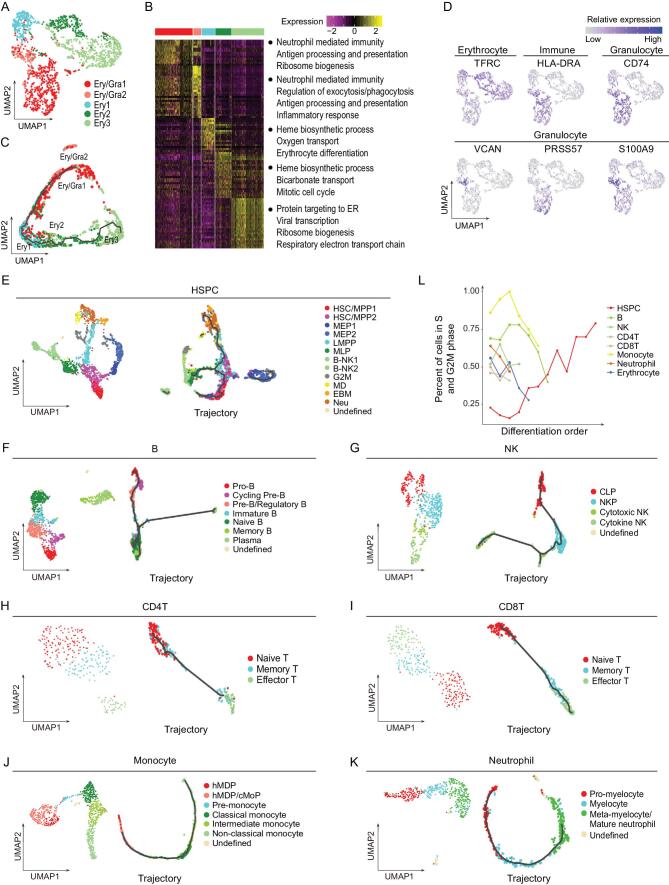
Immune activation of CD74^+^ nucleated erythrocytes and elaborate atlas for other hematopoietic cell populations. (A) UMAP displays the transcriptional clusters for erythrocytes. Colors correspond to cell clusters. (B) Heat map shows the relative expression of the top 50 signature genes for each cluster. Significantly enriched biological processes for corresponding clusters are presented on the right. (C) The differentiation trajectory of erythrocytes by pseudotime analysis using Monocle3. (D) UMAP displays of transcription activities for surface markers and signature genes related to erythroid and granulocyte lineages. (E–K) UMAP displays of the transcriptional clusters (on the left by Seurat) and differentiation trajectory (on the right by Monocle3) for HSPCs (E), B cells (F), NK cells (G), CD4 T cells (H), CD8 T cells (I), monocytes (J) and neutrophils (K). Colors correspond to cell clusters. (L) Percentages of single cells in S and G2M phase for each cell population by line chart.

We next aimed to precisely dissect the differentiation trajectory and characteristics for each particular cell population such as HSPCs, B cells, NK cells, T cells, monocytes, neutrophils and erythrocytes [19]. The batch effects and individual diversities were corrected well for each cell population (Supplementary Fig. 5A). Considering that the immune role of CD71^+^ erythrocytes was reported in previous research [20], first, to illuminate the sub-clusters of continuously sorted erythrocytes, cells from different donors were integrated and followed by dimension reduction and visualization by UMAP. Notably, within the erythroid lineage, we found that Ery/Gra1 and Ery/Gra2 showed highly expressed genes associated with neutrophil and monocyte/dendritic (MD) signatures (Fig. 4A and Supplementary Fig. 5B and C). GO terms of specifically expressed genes in Ery/Gra1 and Ery/Gra2 were enriched on neutrophil-related, phagocytosis and inflammatory responses, along with antigen processing and presentation, further implying the innate and adaptive immune function of these two erythrocyte subsets (Fig. 4B). Additionally, pseudotime analysis showed that Ery/Gra2 was separated from other erythrocyte clusters because of its unique expression of immune signals, for instance *VCAN* and *S100A9* (Fig. 4C and D). The differentiation trajectory also reflected that Ery/Gra1 and Ery/Gra2 were

 

unipotent erythroid progenitors and did not differentiate into granulocytes (Supplementary Fig. 5D). Furthermore, we found that CD74^+^ nucleated erythrocytes mainly contributed to Ery/Gra1 and Ery/Gra2 clusters (Fig. 4D)*.*

Subsequently, the differentiation trajectories and functional diversities for HSPCs, B cells, NK cells, T cells, monocytes and neutrophils were also elaborated. It was noted that intermediate progenitor clusters such as HSC/MPP2, Pre-B/Regulatory B, NKP and hMDP/cMoP were more heterogeneous by transcriptome analysis (Supplementary Fig. 6A). As expected, the differentiation trajectory of HSPCs was highlighted by the branching of progenitors including lymphoid, myeloid and erythroid/megakaryocyte lineages (Fig. 4E and Supplementary Fig. 5E). Memory B and plasma (cytotoxic NK and cytokine NK) were distributed on two different branches by pseudotime analysis, better reflecting the differentiation trajectory of B and NK cell populations (Fig. 4F and G and Supplementary Fig. 5G and H). Regarding T cell populations, both CD4 T and CD8 T cells could be classified into three clusters, respectively corresponding to naive T, memory T and effector T cells. By contrast, CD4 naive T and memory T cells ordered together while CD8 memory T and effector T cells were closely adjacent to each other (Fig. 4H and I and Supplementary Fig. 5I and J). Sub-clusters for monocytes and neutrophils were profiled and further revealed their differentiation trajectories (Fig. 4J and K and Supplementary Fig. 5K and L). In addition, pseudotime analysis based on lncRNAs displayed that lncRNAs could reflect the differentiation trajectories comparable to protein-coding genes (Supplementary Fig. 7). Next, signature genes of each transcriptional cluster were revealed to further support the annotation of blood cell types (Supplementary Fig. 6B, Supplementary Table 1). *AVP* and *CRHBP* were specifically expressed in HSC/MPPs. *CD79A*, *CD79B* and *VPREB1/3* exhibited high specificity and expression in B cells and their progenitors. *NKG7* and *GZMK* were highly expressed in both NK cells and T cells, while *LYZ* and *S100A6/8/9* were highly expressed in both neutrophils and monocytes (Supplementary Fig. 6B). Finally, we found that the cell cycle was activated during HSPC differentiation, while inactivated during blood cell maturation (Fig. 4L). In conclusion, we elaborated the differentiation atlas of each cell population and observed the immune activation of nucleated erythrocytes.

### A web portal for expression data browsing and blood cell type prediction

A user-friendly web interface for browsing and prediction features has been designed to provide access to the single-cell transcriptome data of human blood cells (http://scrna.sklehabc.com/). The browser page was intended for querying the expression of interested genes in 32 immunophenotypic cell types and 43 transcriptional clusters (Supplementary Fig. 8A). This study enabled users to predict the cell types of hematopoietic cells by implementing two approaches (Scmap [21] and Seurat [22]) and visualize them in the hematopoietic atlas (Supplementary Fig. 8B). We evaluated the prediction power using different single-cell datasets. First, 87% of single cells in our dataset could be correctly annotated. Notably, closely related cell clusters, such as HSPC populations, could also be precisely discriminated (accuracies of most clusters exceeded 90%) by our website (Supplementary Fig. 8C). Next, hematopoietic cells downloaded from the studies of Velten *et al.* [8] and Pellin *et al.* [6] were projected to our atlas of blood cells (ABC); most cell types could be correctly matched. Of note, we observed that immature myeloid progenitor 1 (Im1) and immature myeloid progenitor 2 (Im2) cells were annotated to HSC/MPP, LMPP and G2M, suggesting a higher resolution annotation of these cells (Supplementary Fig. 8D). Additionally, HSC, MPP and CMP from Pellin *et al.* were assigned to HSC/MPP2, in agreement with the fact that a low number of HSCs could be harvested by a wider and continuous gating of progenitor cells (Supplementary Fig. 8E). These results indicated that we could better define the cell types by implementing a comprehensive transcriptome reference of human blood cells.

## DISCUSSION

In summary, we established a comprehensive transcriptome reference of the human blood system by combining FACS sorting of 32 immunophenotypically distinct cell types and deep single-cell RNA sequencing. Both gene expression signatures and transcription factor regulatory networks were defined in each cell type. We provided a new angle of lncRNAs for depicting the whole hematopoietic differentiation at single-cell resolution. The in-depth transcriptome data further enabled the discovery of immune activation of nucleated erythrocyte subsets. Finally, we offered an interactive web interface for accessing the atlas data and utilities for cell type prediction.

A growing body of studies has demonstrated that lncRNAs play crucial roles in physiological and pathological processes. Here, we showed that lncRNAs were competitive and consistent with protein-coding genes in distinguishing cell populations. lncRNAs specifically expressed in hematopoietic cells were identified, and their adjacent protein-coding genes were enriched with canonical hematopoietic signatures, implying their potential roles in hematopoietic differentiation. Additionally, signature lncRNAs preferentially orchestrated their adjacent signature protein-coding genes for more differentiated cell types, although this must be further validated by experiments. However, genes adjacent to signature lncRNAs were more general and stochastic for hematopoietic progenitors. This phenomenon supported the point that stem and progenitor cells exhibited more transcriptomic stochasticity [23]. These results provided novel clues for studies of lncRNA biology in human hematopoietic cells.

We thoroughly constructed the differentiation trajectories (with no functional validations) and further defined the signature genes associated with HSPCs, B cells, NK cells, T cells, monocytes, neutrophils and erythrocytes. Notably, we observed a small part of nucleated erythrocytes unexpectedly expressing signatures related to innate immune (neutrophil, phagocytosis and inflammatory response) and adaptive immune (antigen processing and presentation) reactions. It has been reported that nucleated erythrocytes of human cord blood suppressed the production of inflammatory cytokines from monocytes in a lipopolysaccharide (LPS)-mimicked system, to avoid a vigorous innate immune reaction [24]. Our results from adult bone marrow further confirmed the mediator roles of nucleated erythrocytes in immune responses. Additionally, immune roles of CD71^+^ erythrocytes were observed in previous research [20]. Furthermore, by means of single-cell RNA-seq technology, we found that only a small part of CD71^+^ erythrocytes (that is, CD74^+^ erythrocytes) expressed the signature related to immune response.

Our work paves the way for an in-depth understanding of hematopoiesis. The transcription atlas of human blood cells provides a valuable reference for guiding exploration of human physiological and pathological hematopoiesis.

## METHODS

### Sources of donors

Human blood cells including CD34^+^ HSPCs, B cells, NK cells, T cells, monocytes, neutrophils and erythrocytes, were derived from the bone marrow and peripheral blood (only regulatory B, naive B, memory B, cytotoxic NK, cytokine NK and T cells were derived from peripheral blood) of 21 healthy adult donors. All experiments were implemented in accordance with the protocols approved by the institutional ethics review boards from the Institute of Hematology and Blood Diseases Hospital, Chinese Academy of Medical Sciences and Peking Union Medical College. Written informed consents were acquired before sample collection. Donors for CD34^+^ HSPCs were enrolled from Qilu Hospital of Shangdong University and the Institute of Hematology and Blood Disease Hospital. Donors for B cells, NK cells, T cells, monocytes, neutrophils and erythrocytes were obtained from the Institute of Hematology and Blood Disease Hospital.

### Processing of single-cell RNA-seq data

Raw reads of STAT-seq were first split based on the cell-specific barcode sequences attached in read2, and UMI sequence in read2 was integrated into the paired read1 by Python scripts. Then, the template switch oligo (TSO) sequence, the polyA tail sequence and the low-quality reads (N > 10%) were discarded for each separated single cell using Cutadapt (version 1.18) [25]. Subsequently, we aligned the trimmed reads to human reference genome (hg38 from Ensembl) by Hisat2 (version 2.0.3-beta) [26]. Uniquely mapped reads were calculated through htseq-count (version 0.9.1) [27], based on the genome annotations from Ensembl (release 84, hg38) for protein-coding genes and from NONCODE (version v5.0, hg38) [18] for lncRNAs. Finally, the read count of a given gene was quantified by the total number of distinct UMIs, and the raw UMIs of protein-coding genes were normalized by log2(TPM/10 + 1) (TPM: transcripts per million) for downstream analysis. To filter low-quality single cells, mapping rates under 10% for HSPCs/monocytes, under 5% for B cells/NK cells/neutrophils/erythrocytes and under 2.5% for T cells were discarded. Simultaneously, we only retained the cells where more than 1000 protein-coding genes/500 lncRNAs were detected, resulting in 7551 cells for protein-coding genes and 7192 cells for lncRNAs [28–31].

## DATA AVAILABILITY

The single-cell RNA sequencing data reported in this study are deposited in NCBI’s Gene Expression Omnibus (GEO) with the accession numbers GSE137864 and GSE149938. All other relevant data and custom code are available from the corresponding authors upon reasonable request.

## Supplementary Material

nwaa180_Supplemental_Files
