# Supplementary material for: Single-cell transcriptomic landscape of human blood cells
Source: Natl Sci Rev. 2020 Aug 24;8(3):nwaa180. doi: 10.1093/nsr/nwaa180 (PMC8288407; doi:10.1093/nsr/nwaa180)
Supplement: nwaa180_Supplemental_Files [file nwaa180_supplemental_files.zip › Supplementary_File.docx]

**Single-cell Transcriptomic Landscape of Human Blood Cells**

**Methods**

**Sample preparation and FACS sorting**

Mononuclear cells from bone marrow and peripheral blood were isolated by Ficoll-Paque (GE Healthcare) density gradient centrifugation for FACS sorting, except that mononuclear cells of neutrophils were prepared by ammonium chloride solution (STEMCELL). Mononuclear cells were freshly prepared for HSPCs, B cells, NK cells, T cells and neutrophils, or cryopreserved in 10% DMSO in liquid nitrogen (-196℃) until use for monocytes and erythrocytes. Frozen cells were quickly thawed and stained with human antibodies. For erythrocytes, CD45^-^ cells were enriched from mononuclear cells by CD45 microbead kit (Miltenyi Biotec) before staining with antibodies. After incubating on ice for 30 minutes in the dark, samples were washed with PBS + 2% FBS, and DAPI (1 μg/ml, Sigma-Aldrich) or 7-AAD (BD Biosciences) were added to cell suspensions to deplete dead cells. Based on FACS sorting (BD FACSAria III, BD Bioscience), different HSPCs were isolated including HSC (Lin^-^CD34^+^CD38^-^CD45RA^-^CD90^+^CD49f^+^CD10^-^), MPP (Lin^-^CD34^+^CD38^-^CD45RA^-^CD90^-^CD10^-^), CMP (Lin^-^CD34^+^CD38^+^CD10^-^CD45RA^-^CD135^+^), MEP (Lin^-^CD34^+^CD38^+^CD10^-^CD45RA^-^CD135^-^), LMPP (Lin^-^CD34^+^CD38^-^CD45RA^+^CD90^-^CD10^-^), MLP (Lin^-^CD34^+^CD38^-^CD45RA^+^CD90^-^CD10^+^), BNK (Lin^-^CD34^+^CD38^+^CD45RA^+^CD10^+^) and GMP (Lin^-^CD34^+^CD38^+^CD10^-^CD45RA^+^CD135^+^). B cell populations were isolated including proB (CD34^+^CD10^+^CD19^+^IgM^-^), preB (CD34^-^CD10^+^CD19^+^IgM^-^), immature B (CD34^-^CD10^+^CD19^+^IgM^+^), regulatory B (CD19^+^CD24^high^CD38^high^), naive B (CD19^+^CD24^int^CD38^int^), memory B (CD19^+^CD24^+^CD38^low^/^-^CD27^+^) and plasma (CD14^-^CD2^-^CD38^+^CD138^+^). NK cell populations included CLP (Lin^-^CD34^+^CD38^+^CD123^-^CD45RA^+^CD7^+^CD10^+^CD127^+^), NKP (Lin^-^CD34^+^CD38^+^CD123^-^CD45RA^+^CD7^+^CD10^+^CD127^-^), cytotoxic NK (CD3^-^CD56^dim^CD16^+^) and cytokine NK (CD3^-^CD56^bright^CD16^low^). T cell populations contained CD4 T (CD3^+^CD4^+^) and CD8 T (CD3^+^CD8^+^) cells. Monocytes included hMDP (Lin^-^CD34^+^CD38^high^CD10^-^CD45RA^+^CD123^int^CD115^+^CD116^-^), cMoP (Lin^-^CD34^+^CD38^+^CD10^-^CD45RA^+^CD123^low^CD135^+^CLEC12A^high^CD64^high^), pre-monocyte (Lin^-^CD34^-^CD38^+^CD10^-^CD45RA^high^CD123^low^CD135^+^CLEC12A^high^CD64^high^), classical monocyte (CD45^+^Lin^-^(CD3, CD19, CD56)CD14^++^CD16^-^), intermediate monocyte (CD45^+^Lin^-^(CD3, CD19, CD56)CD14^++^CD16^+^) and non-classical monocyte (CD45^+^Lin^-^(CD3, CD19, CD56)CD14^+^CD16^++^). Neutrophils contained pro-myelocyte (CD45^+^CD33^+^CD11b^-^CD16^-^), myelocyte (CD45^+^CD33^+^CD11b^low^CD16^-^), meta-myelocyte (CD45^+^CD33^+^CD11b^+^CD16^low^) and mature neutrophil (CD45^+^CD33^+^CD11b^+^CD16^+^). Erythrocytes were continuously sorted by CD235a, BAND3 and α4-intergrin.

The following antibodies for HSPCs were used: lineage cocktail (CD3, CD14, CD16, CD19, CD20, CD56) (BV510, Biolegend), CD34 (APC, clone 581, BD Biosciences), CD38 (FITC, clone HB7, BD Biosciences), CD90 (PerCP/Cyanine5.5, clone 5E10, BD Biosciences), CD45RA (PE/Cy7, clone HI100, Biolegend), CD49f (BV605, clone GoH3, BD Biosciences), CD10 (BV786, clone HI10a, BD Biosciences) and CD135 (PE, clone 4G8, BD Biosciences). Antibodies for B cells are summarized as follows: CD10 (PE, clone H110a, Biolegend), CD19 (PerCP-cy5.5, clone HIB19, Biolegend), CD27 (PE-Cy7, clone O323, Biolegend), IgM (Alexa Fluor647, clone MHM-88, Biolegend), CD38 (APC, clone HIT2, BD), CD2 (PerCP-cy5.5, clone RPA-2.10, BD), CD14 (APC-cy7, clone MφP9, BD), CD24 (PE, clone ML5, Biolegend), CD138 (PE, clone DL-101, Biolegend) and CD34 (FITC, clone 8G12, BD). NK cells: lineage cocktail (CD3, CD14, CD16, CD19, CD20, CD56) (FITC, BioLegend), CD34 (APC, clone 4H11, Invitrogen), CD38 (PE-Cy7, clone HB7, Invitrogen), CD123 (PE, clone 7G3, BD Biosciences), CD45RA (APC-Cy7, clone HI100, BioLegend), CD7 (BV711, clone M-T701, BD Biosciences), CD10 (BV510, clone HI10a, BD Biosciences), CD127 (PerCP-Cy5.5, clone A019D5, BioLegend), CD3 (FITC, clone UCHT1, BD Biosciences), CD56 (APC, clone MEM-188, BioLegend) and CD16 (PE, clone eBioCB16, eBioscience). T cells: CD3 (APC-CyTM7, clone SK7, BD Biosciences), CD4 (APC, clone RPA-T4, BD Biosciences) and CD8 (PE-CyTM7, clone RPA-T8, BD Biosciences). Monocytes: lineage cocktail (CD3, CD14, CD16, CD19, CD20, CD56) (FITC, BioLegend), CD34 (APC, clone 581, BD Biosciences), CD38 (BV510, clone HB7, Biolegend), CD10 (BV786, clone HI10a, BD Biosciences), CD123 (PE/CY7, clone 6H6, Biolegend), CD45RA (APC/CY7, clone HI100, Biolegend), CD135 (PE, clone 4G8, BD Biosciences), CD64 (BV605, clone 10.1, Biolegend), CLEC12A (PerCP/Cyanine5.5, clone 50C1, Biolegend), CD116 (PE, clone 4H1, Biolegend), CD115 (PerCP/Cyanine5.5, clone 9-4D2-1E4, Biolegend), CD45 (APC, clone HI30, BD Biosciences) and CD14 (FITC, clone M5E2, BD Biosciences). Neutrophils: CD45 (FITC, clone HI30, BD Biosciences), CD33 (PerCP-cy5.5, clone P67.6, BD Biosciences), CD11b (APC-cy7, clone  ICRF44, BD Biosciences) and CD16 (PE, clone 3G8, BD Biosciences). CD235a (FIFC, clone HIR2, BD Biosciences), CD49d (PE, clone 9F10, Biolgend) and Band3 for erythrocytes were obtained from Xiuli An.

**Library construction and sequencing**

Single-cell library construction was performed referring to the STAT-seq protocol (1-2) with some modifications(3). Lysis buffer (total volume 2.55 μl) including recombinant ribonuclease inhibitor (40U/μl, TAKARA), 10% Triton X-100 (Sigma-Aldrich), dNTP (10 mM, TAKARA), nuclease-free water (Ambion) and barcode primers (10 μM, synthesis by Invitrogen), was prepared in advance and reserved at -80 ℃. First, single cells were obtained by flow cytometry into 0.2 mL thin-wall 8 strip PCR tubes holding 2.55 μl cell lysis buffer. To connect the cell-specific barcode sequence and unique molecular identifier (UMI) to the 3’end of RNA, reverse transcription was conducted at 25 °C 5 min, 42°C 60 min, 50°C 30 min, 72°C 10 min and 4°C hold. Then, 20 cycles of PCR were applied to amplify cDNA, the products of which were merged for purification by way of DNA Clean and Concentrator kit (Zymo Research) and 0.8X Agencourt AMPure XP beads (Beckman Coulter). An additional 4 cycles of PCR were implemented to anchor the biotin index to the 3’ ends of PCR products. Dynabeads MyOne Streptavidin C1 (Invitrogen) was adopted for the biotin enrichment of shared cDNA (~300 bp by Focused-ultrasonicator, Covaris M220). Finally, the cDNA libraries were generated by a KAPA Hyper Prep Kit (Kapa Biosystems) and sequenced on the Illumina HiSeq4000 platform as paired-end 150bp reads (Novogene).

**Dimension reduction and clustering analysis**

The Seurat package (version 3.0.2)(4) implemented in R (version 3.6.0) was used to remove batch effects, reduce dimension and cluster cells based on the UMI count. First, “SCTransform”(5) was applied to process data before reducing dimension for the transcriptional atlas of all blood cells, while “IntegrateData” was adopted to anchor samples from different donors for each particular cell population with the default 2000 features. Then, “RunPCA” was performed based on the union of highly variable genes calculated by “FindVariableFeatures”, and single cells were projected into two-dimension space using “RunUMAP”(6-7). Finally, we applied “FindNeighbors” and “FindClusters” to cluster single cells at the resolution of 0.6, 0.45, 0.31, 0.7, 0.6, 0.55, 0.15 and 0.22 respectively for HSPCs, B cells, NK cells, CD4 T cells, CD8 T cells, monocytes, neutrophils and erythrocytes, and visualize them by UMAP. The detailed cell-donor information, cell to cluster associations and cell pseudotime prediction of all single cells are provided in Supplementary Table 2.

**Identification of signature genes and DEGs**

“FindAllMarkers” from the Seurat package was used to detect cell type/cluster-specific signature genes, while “FindMarkers” was applied to identify the DEGs between any two given groups. AUC scores of signature genes obtained by pROC represented the specificity of each gene for each cluster (ranging from 0 to 1; the larger the score, the more unique to each cluster). The filtered criteria for signature genes and DEGs was fold-change ≥ 1.5 or ≤ 0.67 and adjusted P-value ≤ 0.05. Gene ontology enrichment analysis on genes was achieved by Enrichr(8).

**Transcription factor regulatory network analysis**

SCENIC(9), a software to construct the transcription factor regulatory network based on single-cell RNA-seq data, was used to explore the regulatory landscape underlying the hematopoietic system. Human transcription factors were extracted from the SCENIC package in R version. Utilizing the “grn”, “ctx” and “aucell” algorithms from SCENIC software, we acquired the regulon activity scores (RASs) of enriched transcription factors in each single cell. Then, the RAS matrix of all single cells was submitted to Seurat to investigate the hematopoietic differentiation in regulation view. Meanwhile, the transcription factors with high RAS for each regulatory cluster were identified. To further verify the hematopoietic differentiation in regulation view, SPRING(10) was also applied to repeat the reducing dimension and visualization analysis. Compared with known transcription factors, combined with gene ontology analysis and motif analysis by HOMER(11), novel regulons for each cell population could also be determined. The annotated motif sequences of transcription factors were downloaded from JASPAR(12) database. Finally, regulatory networks comprised of transcription factors and their target genes were displayed by Cytoscape(13) software.

**Analysis of lncRNAs**

Referring to a previous report(14), neighboring protein-coding genes with distances less than 5kb were scanned along the genome for signature lncRNAs by bedtools (version 2.25.0)(15). Co-expression associations of lncRNA and the distal protein-coding genes (distances more than 5kb) were built by the “grn” algorithm from SCENIC.

**Pseudotime analysis by Monocle3 and scanpy**

On the basis of UMI count matrix created respectively for HSPCs, B cells, NK cells, T cells, monocytes, neutrophils and erythrocytes, pseudotime trajectory for each was constructed by Monocle3 (version 0.2.0)(16-17). First, “preprocess” and “align_cds” were applied to pre-process data and remove batch effects among different donors. Then, dimension reduction was performed by “reduce_dimension” using UMAP. Finally, pseudotime trajectory of newly identified cell clusters for each population was visualized, followed by “cluster_cells” and “learn_graph”. Meanwhile, another package named scanpy(18) was used to complement the pseudotime analysis based on “ForceAtlas2”.

**Cell cycle analysis**

Based on the log2(TPM/10 + 1) value of protein-coding genes, single cells were designated into G1, S or G2M phase by scran package (version 1.10.2)(19). 43 genes related to G1/S and 55 genes related to G2/M(20) were taken into the calculation of G1/S and G2/M score, and G0 phase was extracted from G1 phase when the sum score of G1/S and G2/M was less than 3 according to the density distribution of scores.

**Statistical analysis, plots and websites**

Nonparametric Mann–Whitney test was carried out by R language to compare the difference between two groups. Reported P-values were from two-sided tests and P-value < 0.05 was considered to be significant. R program was used to plot heatmaps (by pheatmap), boxplots, barplots, etc. (by ggplot2). The website was compiled and accomplished by R Shiny. Both Scmap and Seurat were used to achieve blood cell type prediction. Note that the visualization plot in the website was generated by Scmap. The R package of blood cell type prediction by both Scmap and Seurat is available at <https://github.com/pzhulab/abcCellmap>.

**References**

1. Picelli, S, Bjorklund, AK, Faridani, OR*, et al.* Smart-seq2 for sensitive full-length transcriptome profiling in single cells. *Nat Methods*. 2013; **10**(11): 1096-8.

2. Picelli, S, Faridani, OR, Björklund, ÅK*, et al.* Full-length RNA-seq from single cells using Smart-seq2. *Nature protocols*. 2014; **9**(1): 171.

3. Li, L, Dong, J, Yan, L*, et al.* Single-Cell RNA-Seq Analysis Maps Development of Human Germline Cells and Gonadal Niche Interactions. *Cell Stem Cell*. 2017; **20**(6): 891-2.

4. Stuart, T, Butler, A, Hoffman, P*, et al.* Comprehensive Integration of Single-Cell Data. *Cell*. 2019; **177**(7): 1888-902 e21.

5. Hafemeister, C, Satija, R. Normalization and variance stabilization of single-cell RNA-seq data using regularized negative binomial regression. *Genome Biology*. 2019; **20**(1): 1-15.

6. Becht, E, McInnes, L, Healy, J*, et al.* Dimensionality reduction for visualizing single-cell data using UMAP. *Nature biotechnology*. 2019; **37**(1): 38.

7. McInnes, L, Healy, J, Melville, J. Umap: Uniform manifold approximation and projection for dimension reduction. *arXiv preprint arXiv:180203426*. 2018.

8. Kuleshov, MV, Jones, MR, Rouillard, AD*, et al.* Enrichr: a comprehensive gene set enrichment analysis web server 2016 update. *Nucleic Acids Res*. 2016; **44**(W1): W90-7.

9. Aibar, S, Gonzalez-Blas, CB, Moerman, T*, et al.* SCENIC: single-cell regulatory network inference and clustering. *Nat Methods*. 2017; **14**(11): 1083-6.

10. Weinreb, C, Wolock, S, Klein, AM. SPRING: a kinetic interface for visualizing high dimensional single-cell expression data. *Bioinformatics*. 2018; **34**(7): 1246-8.

11. Heinz, S, Benner, C, Spann, N*, et al.* Simple combinations of lineage-determining transcription factors prime cis-regulatory elements required for macrophage and B cell identities. *Mol Cell*. 2010; **38**(4): 576-89.

12. Mathelier, A, Fornes, O, Arenillas, DJ*, et al.* JASPAR 2016: a major expansion and update of the open-access database of transcription factor binding profiles. *Nucleic Acids Res*. 2016; **44**(D1): D110-5.

13. Shannon, P, Markiel, A, Ozier, O*, et al.* Cytoscape: a software environment for integrated models of biomolecular interaction networks. *Genome Res*. 2003; **13**(11): 2498-504.

14. Zhou, J, Xu, J, Zhang, L*, et al.* Combined Single-Cell Profiling of lncRNAs and Functional Screening Reveals that H19 Is Pivotal for Embryonic Hematopoietic Stem Cell Development. *Cell Stem Cell*. 2019; **24**(2): 285-98 e5.

15. Quinlan, AR, Hall, IM. BEDTools: a flexible suite of utilities for comparing genomic features. *Bioinformatics*. 2010; **26**(6): 841-2.

16. Qiu, X, Mao, Q, Tang, Y*, et al.* Reversed graph embedding resolves complex single-cell trajectories. *Nat Methods*. 2017; **14**(10): 979-82.

17. Cao, J, Spielmann, M, Qiu, X*, et al.* The single-cell transcriptional landscape of mammalian organogenesis. *Nature*. 2019; **566**(7745): 496-502.

18. Wolf, FA, Angerer, P, Theis, FJ. SCANPY: large-scale single-cell gene expression data analysis. *Genome Biol*. 2018; **19**(1): 15.

19. Lun, AT, McCarthy, DJ, Marioni, JC. A step-by-step workflow for low-level analysis of single-cell RNA-seq data with Bioconductor. *F1000Res*. 2016; **5**: 2122.

20. Tirosh, I, Venteicher, AS, Hebert, C*, et al.* Single-cell RNA-seq supports a developmental hierarchy in human oligodendroglioma. *Nature*. 2016; **539**(7628): 309-13.

**Supplementary figures and tables**

**Supplementary Fig. 1 | Sorting strategy for human hematopoietic cell types**

**A-J**, Representative display of sorting strategy for bone marrow-derived HSPCs (**A**), T cells (**B**), NK cells (**C**), B cells (**D**), monocytes (**E**), neutrophils (**F**) and erythrocytes (**G**), along with peripheral blood-derived T cells (**H**), NK cells (**I**) and B cells (**J**).

**Supplementary Fig. 2 | Single-cell transcriptional atlas of human hematopoietic cells**

**A**, Sample information for hematopoietic cells. The detailed antibodies for each cell type, along with the number of donors and single cells for each population are shown in the figure. **B**, ABC detects more low-abundance genes than bone marrow samples from HCA. **C**, Transcriptional atlas of 7,551 human hematopoietic cells by UMAP. Colors indicate cell types.

**Supplementary Fig. 3 | Regulatory networks underlie human hematopoietic system**

**A**, Distributions of 32 immunophenotypic cell types based on regulons by SPRING. **B**, Heat map shows the percentages of 32 immunophenotypic cell types in 20 regulatory clusters. **C,D,** Cytoscape shows the regulatory networks underlie neutrophil/monocyte (**C**) and erythroid lineages (**D**).

**Supplementary Fig. 4 | Single-cell transcriptional atlas of human hematopoietic cells based on lncRNAs**

**A**, Transcriptional atlas of 7,192 human hematopoietic cells based on lncRNAs by UMAP. Colors indicate cell types. B, Bar plot shows the proportion of distal signature lncRNAs co-expressed with signature protein-coding genes for each immunophenotypic cell type. **C**, Bar plot shows the overlap between signature lncRNAs adjacent protein-coding genes and cell type specifically expressed genes from Velten *et al*. and HCA.

**Supplementary Fig. 5 | Immune function of nucleated erythrocytes and trajectory analysis for each hematopoietic cell population by scanpy**

**A,** Assessment of batch effects and individual variations. **B**, UMAP displays of transcription activities for erythroid signature genes. **C**, Bar plot shows the overlap between signatures of erythroid cell clusters and cell type specifically expressed genes from Velten *et al*. and HCA. **D**, The differentiation trajectory of erythrocytes and neutrophils revealed by pseudotime analysis using Monocle3. **E-L**, Visualization of the differentiation trajectory by scanpy for HSPCs **(E)**, erythrocytes **(F)**, B cells **(G)**, NK cells **(H)**, CD4 T cells **(I)**, CD8 T cells **(J)**, monocytes **(K)** and neutrophils **(L)**. Colors correspond to cell clusters.

**Supplementary Fig. 6 |** **The signature genes for each hematopoietic cell population**

**A,** Heatmaps display the percentages of immunophenotypic cell types in transcriptional cell clusters for HSPCs, B cells, NK cells, monocytes and neutrophils. **B,** The top 10 signature genes calculated by AUC score in each transcriptional cell clusters for HSPCs, B cells, NK cells, T cells, monocytes, neutrophils and erythrocytes.

**Supplementary Fig. 7 | Elaborate atlas for each hematopoietic cell population based on lncRNAs**

**A-H,** UMAP displays of the transcriptional clusters (left by Seurat) and differentiation trajectory (middle by Monocle3 and right by scanpy) for HSPCs **(A)**, erythrocytes **(B)**, B cells **(C)**, NK cells **(D)**, CD4 T cells **(E)**, CD8 T cells **(F)**, monocytes **(G)** and neutrophils **(H)**. Colors correspond to cell clusters.

**Supplementary Fig. 8 | A web interface for data browse and cell type prediction**

**A**, Data browse interface. **B**, Cell type prediction interface. **C-E**, Heat maps display the percentages of transferred cell clusters in true cell clusters from ABC (**C**), Velten *et al*. (**D**) and Pellin *et al*. (**E**).

**Supplementary Table 1, Information of 43 transcriptional cell clusters and signature genes with AUC score.**

**Supplementary Table 2, Cell to donor information, cell to cluster associations and cell pseudotime prediction associations.**
