# Supplementary figures and images for: Single-cell transcriptomic landscape of human blood cells
Source: Natl Sci Rev. 2020 Aug 24;8(3):nwaa180. doi: 10.1093/nsr/nwaa180 (PMC8288407; doi:10.1093/nsr/nwaa180)

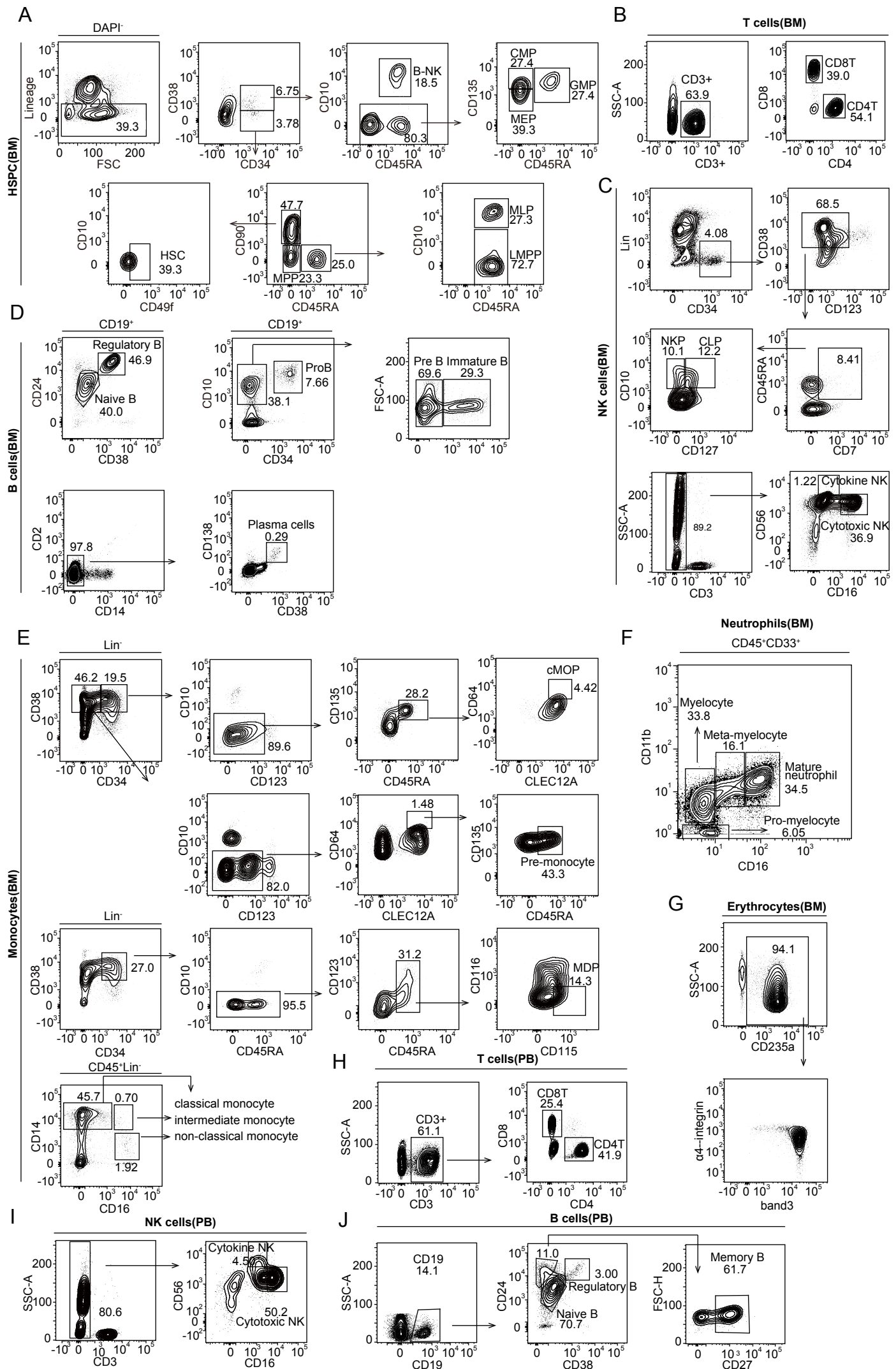

Supplement: nwaa180_Supplemental_Files [file nwaa180_supplemental_files.zip › Supplementary_Fig._1.pdf]

A

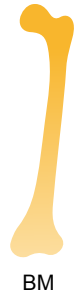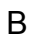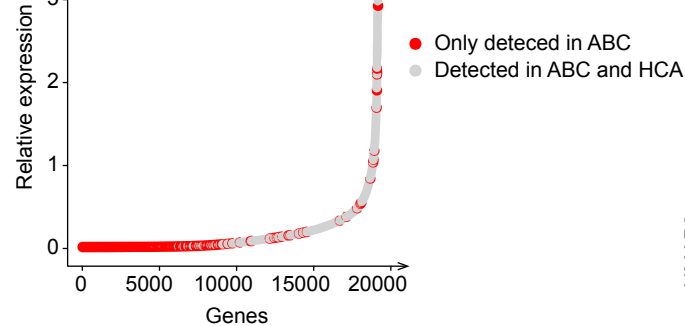

Supplement: nwaa180_Supplemental_Files [file nwaa180_supplemental_files.zip › Supplementary_Fig._2.pdf]

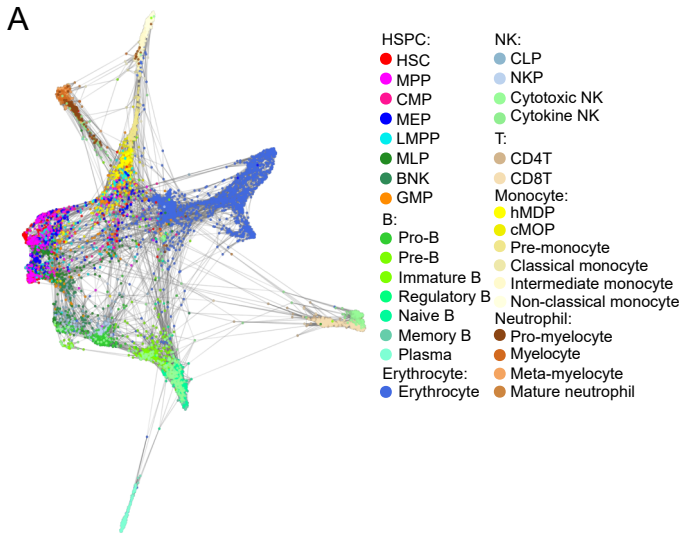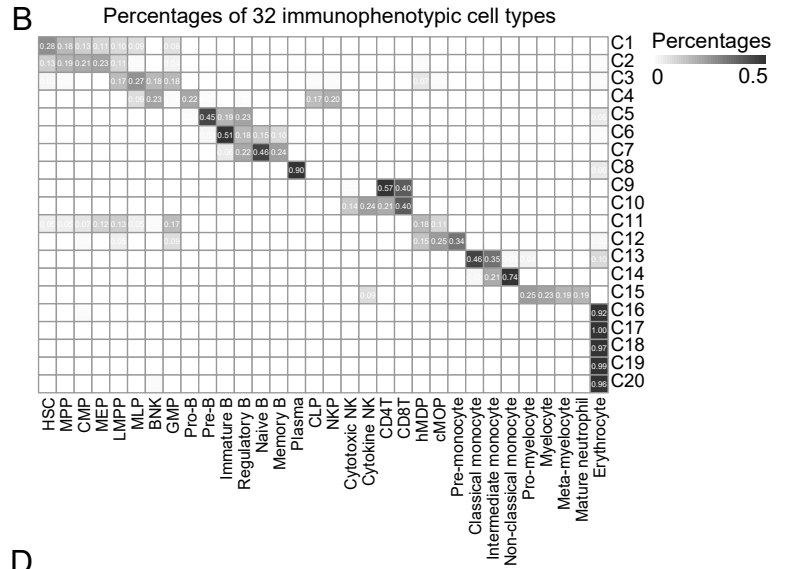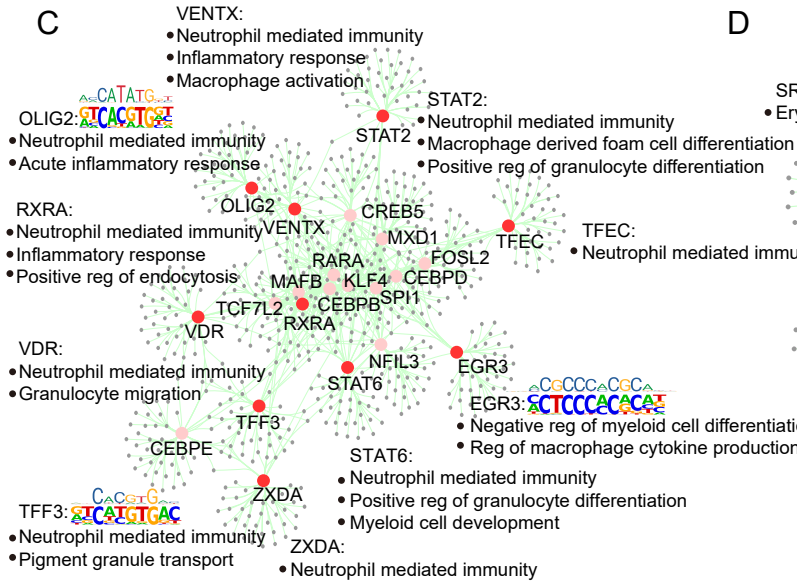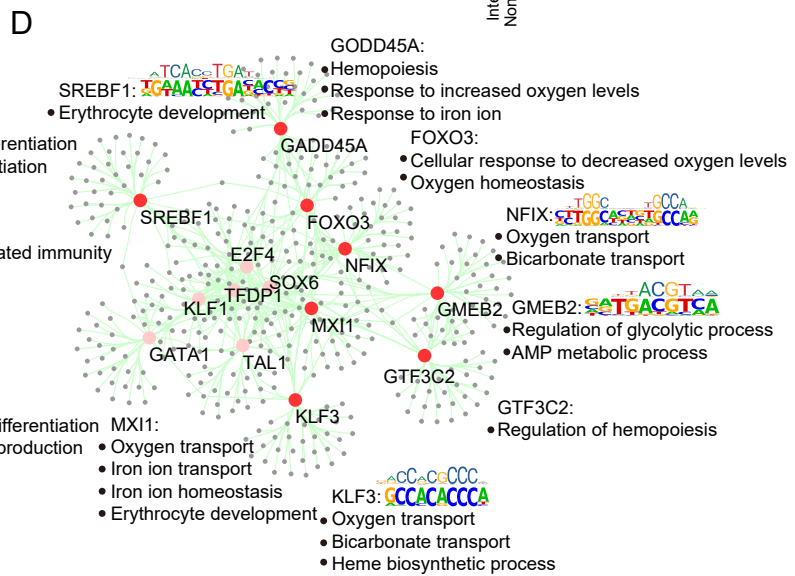

Supplement: nwaa180_Supplemental_Files [file nwaa180_supplemental_files.zip › Supplementary_Fig._3.pdf]

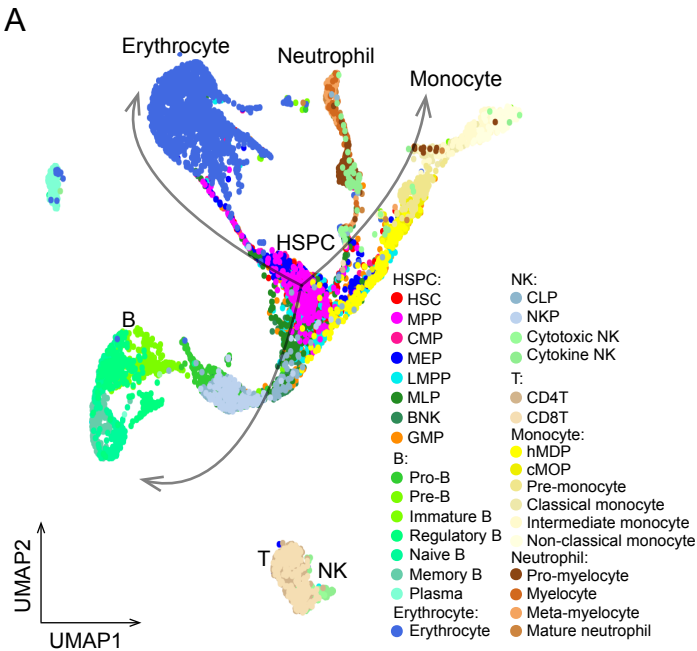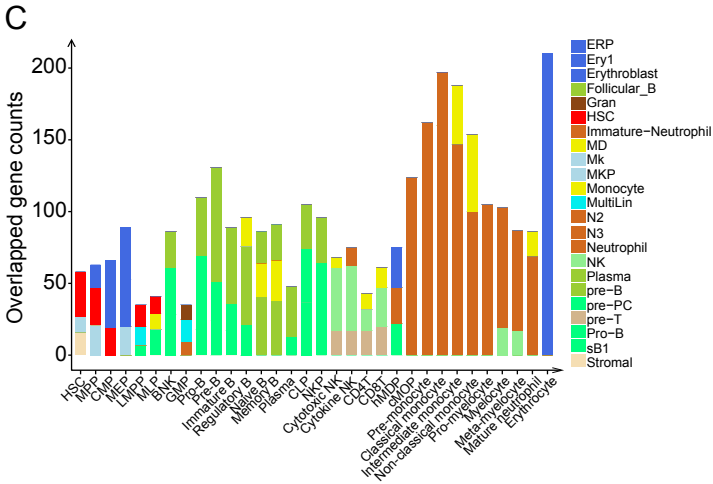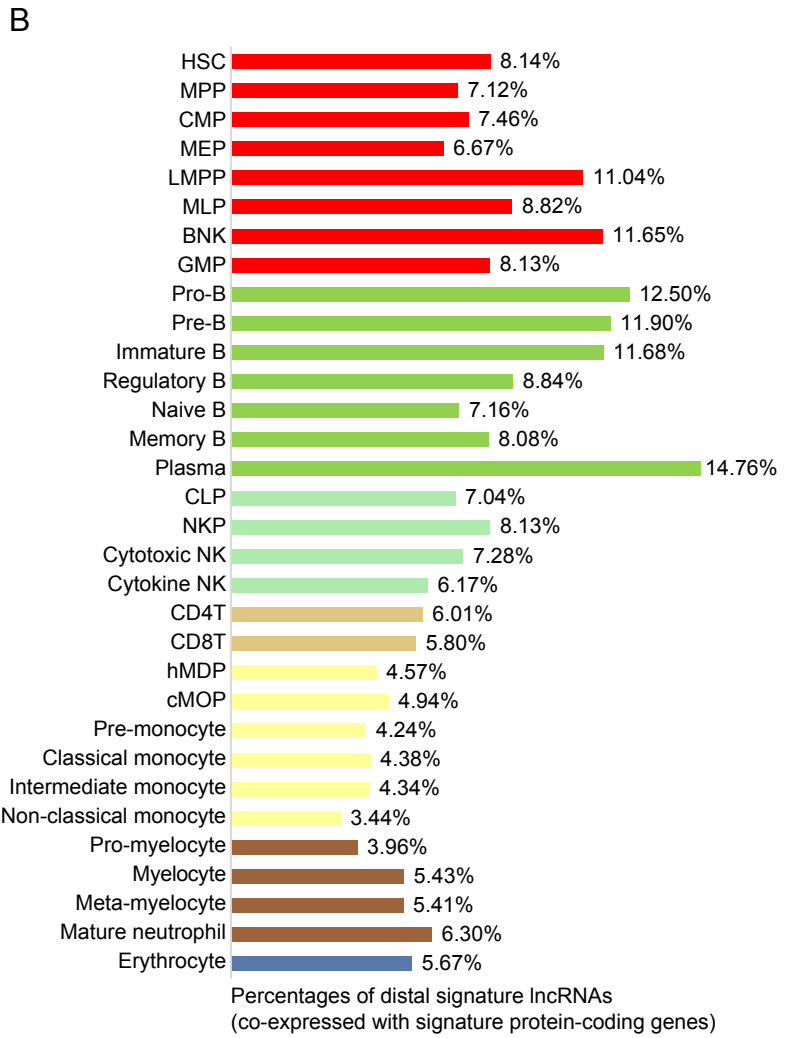

Supplement: nwaa180_Supplemental_Files [file nwaa180_supplemental_files.zip › Supplementary_Fig._4.pdf]

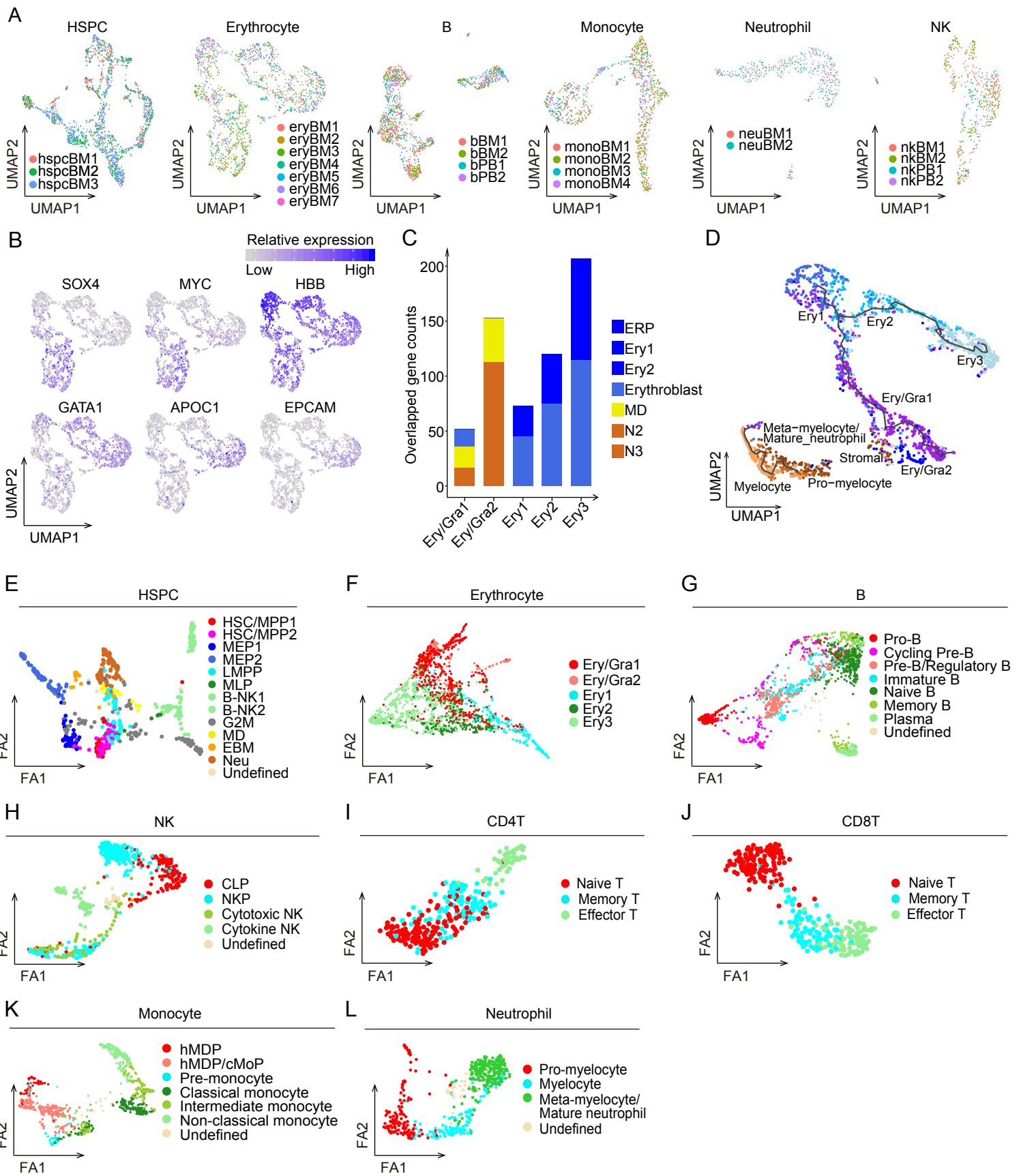

Supplement: nwaa180_Supplemental_Files [file nwaa180_supplemental_files.zip › Supplementary_Fig._5.pdf]

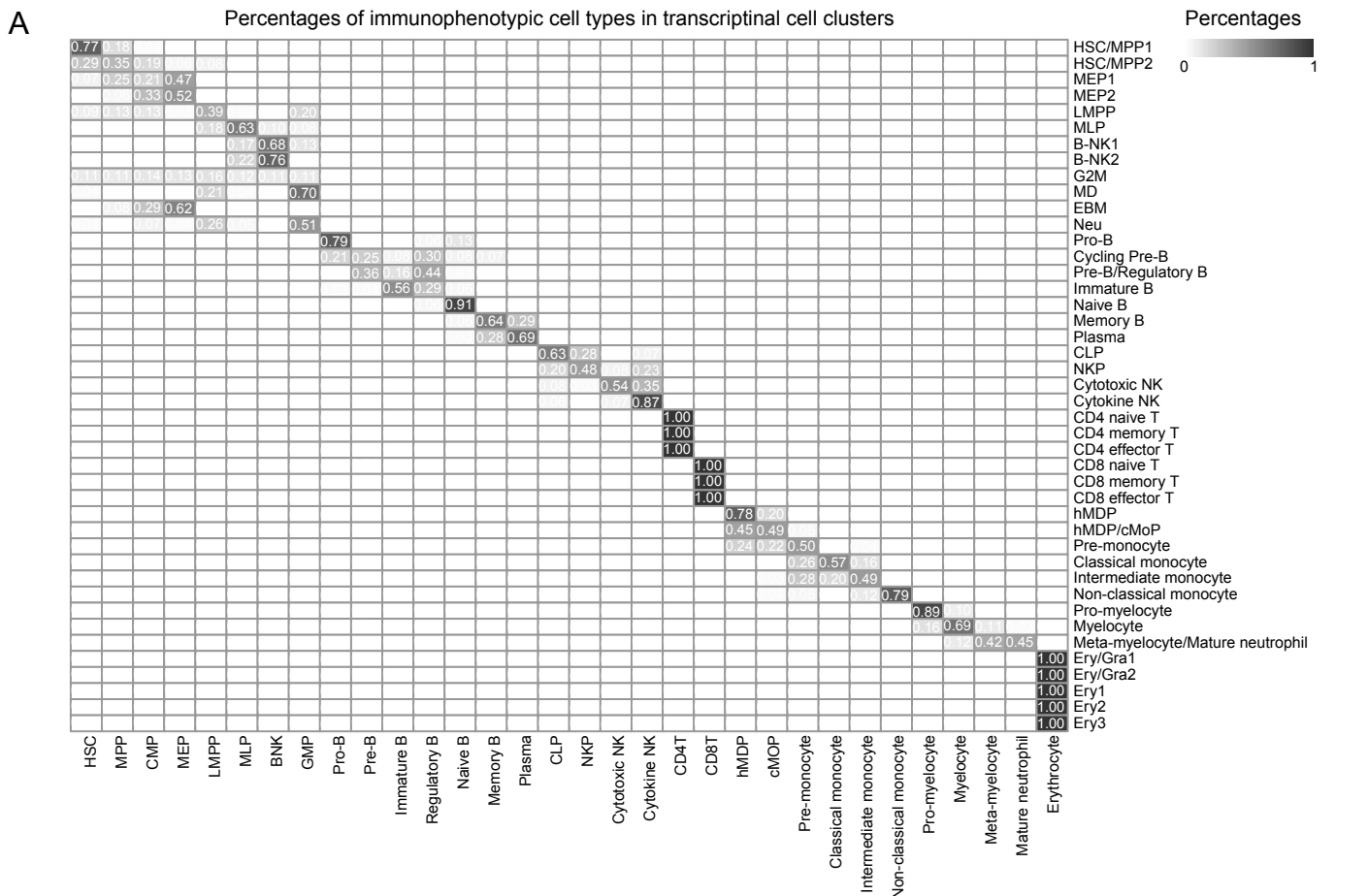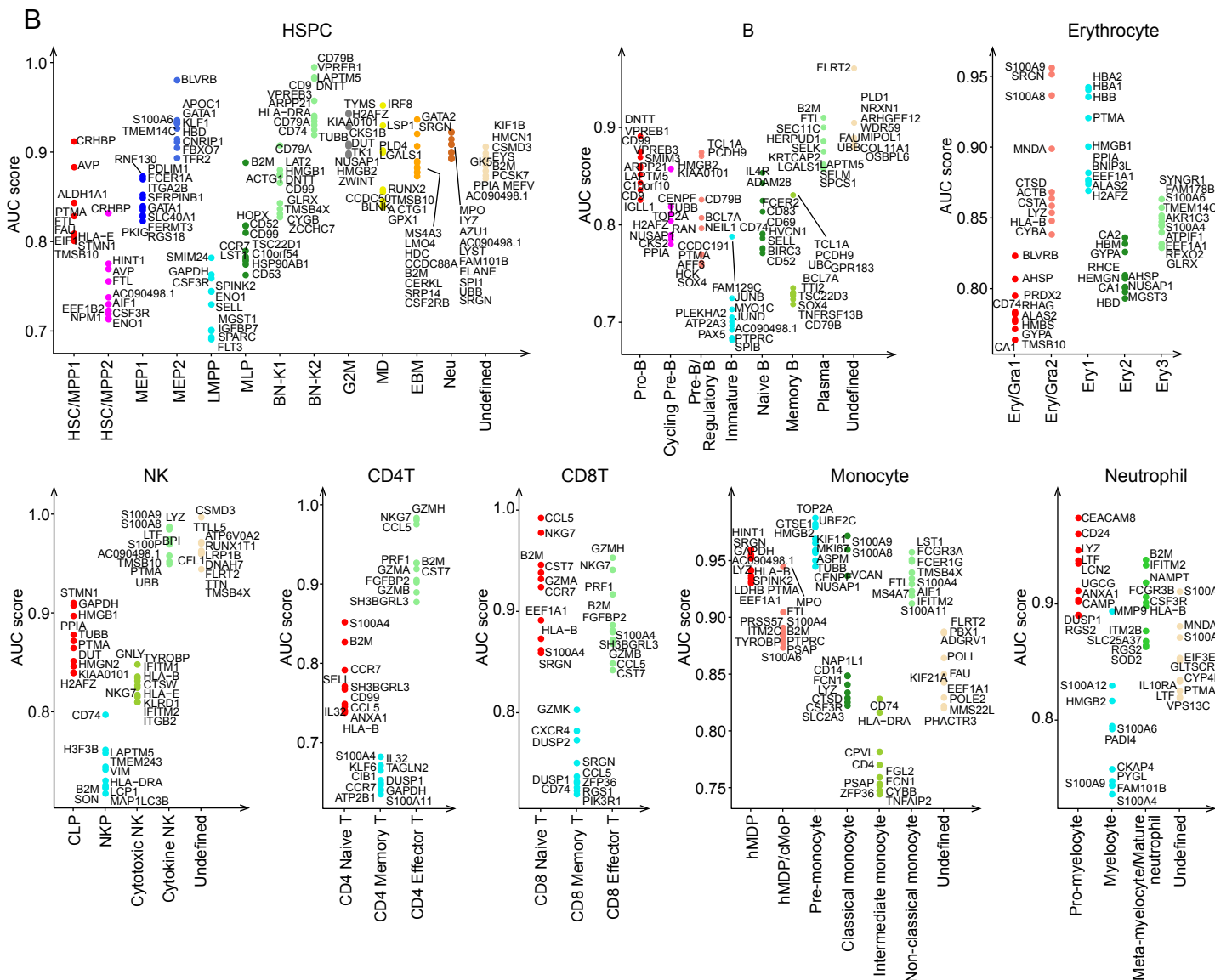

Supplement: nwaa180_Supplemental_Files [file nwaa180_supplemental_files.zip › Supplementary_Fig._6.pdf]

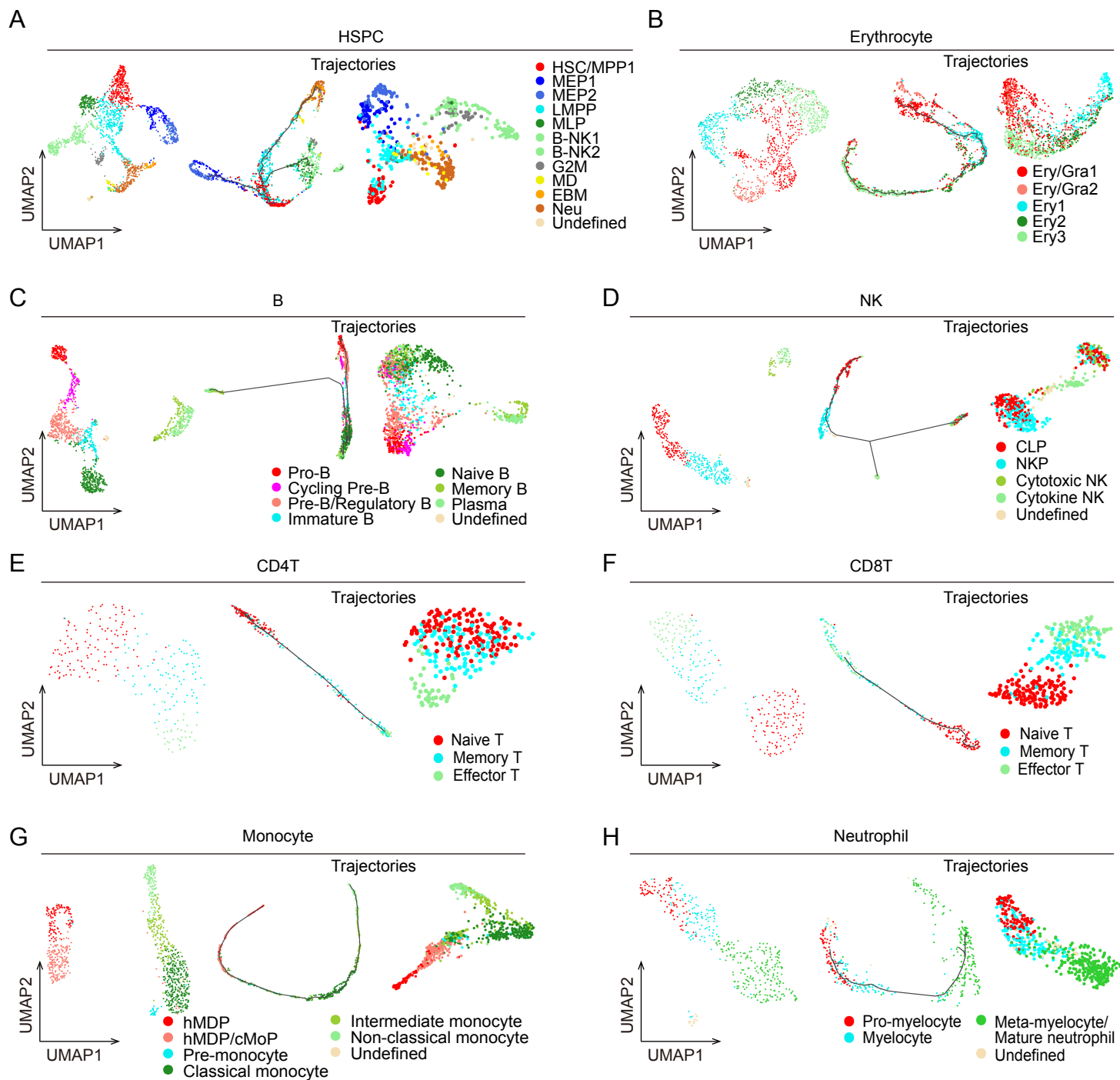

Supplement: nwaa180_Supplemental_Files [file nwaa180_supplemental_files.zip › Supplementary_Fig._7.pdf]

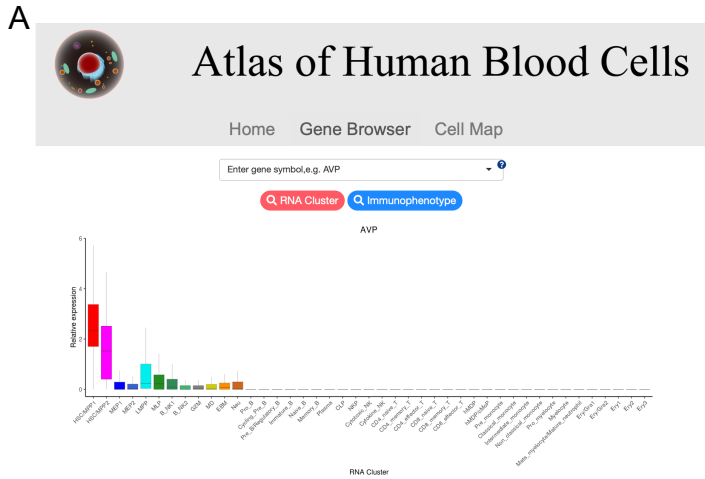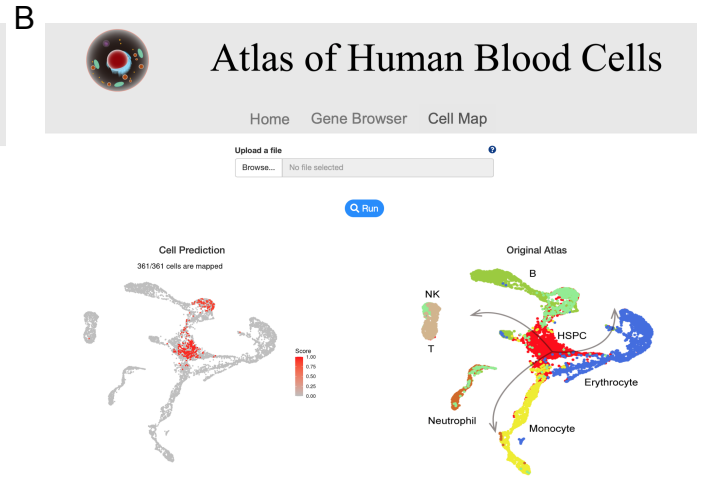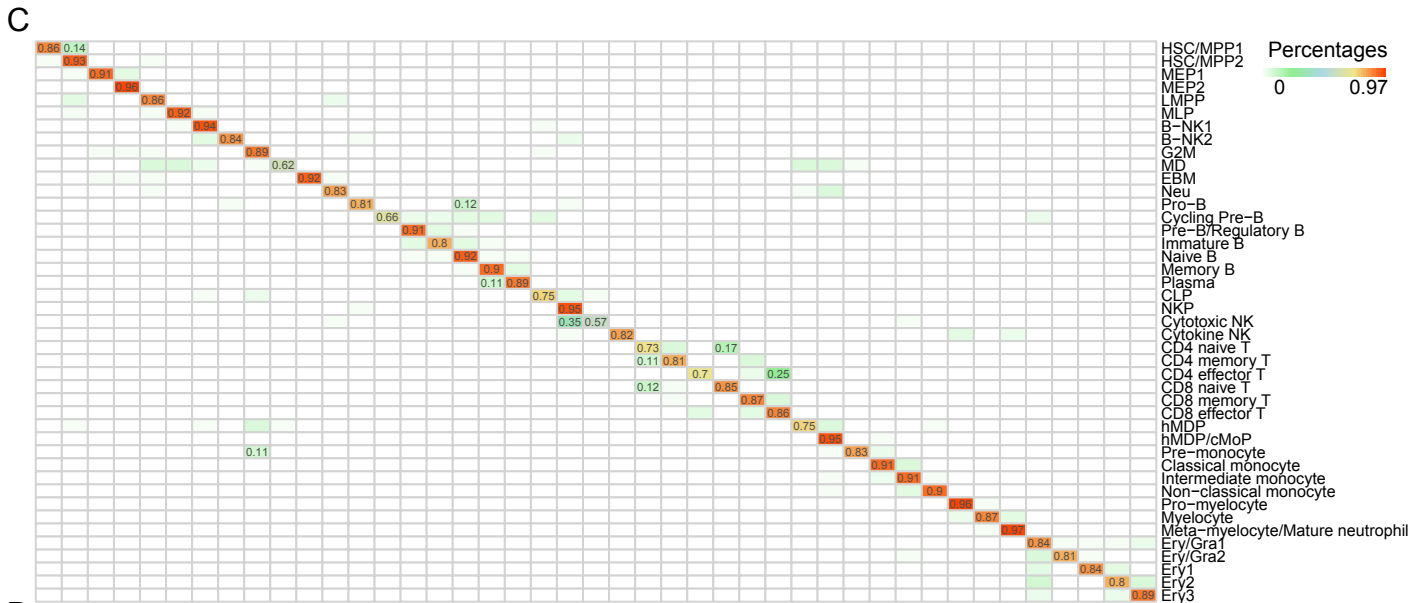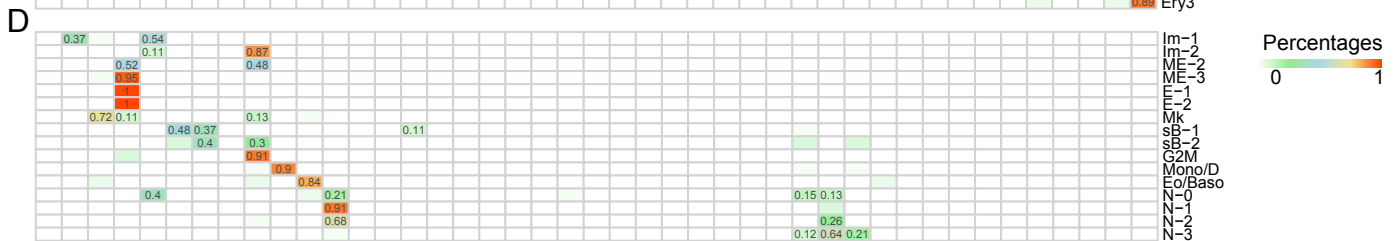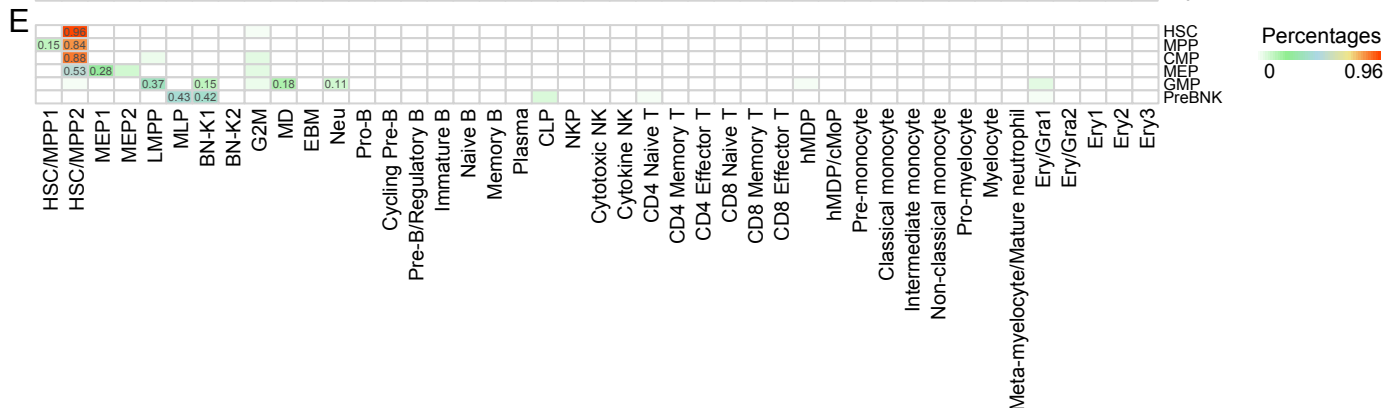

Supplement: nwaa180_Supplemental_Files [file nwaa180_supplemental_files.zip › Supplementary_Fig._8.pdf]
